# Gender difference in the association of dietary patterns and metabolic parameters with obesity in young and middle-aged adults with dyslipidemia and abnormal fasting plasma glucose in Taiwan

**DOI:** 10.1186/s12937-019-0503-x

**Published:** 2019-11-16

**Authors:** Li-Yin Lin, Chien-Yeh Hsu, Hsiu-An Lee, Alexey A. Tinkov, Anatoly V. Skalny, Wan-Hsiang Wang, Jane C.-J. Chao

**Affiliations:** 1 School of Nutrition and Health Sciences, College of Nutrition, Taipei Medical University, 250 Wu-Hsing Street, Taipei, 11031 Taiwan; 2 Department of Information Management, National Taipei University of Nursing and Health Sciences, 365 Ming-Te Road, Peitou District, Taipei, 11219 Taiwan; 3 Master Program in Global Health and Development, College of Public Health, Taipei Medical University, 250 Wu-Hsing Street, Taipei, 11031 Taiwan; 4 Department of Computer Science and Information Engineering, Tamkang University, 151 Yingzhuan Road, Tamsui District, New Taipei, 25137 Taiwan; 5 Yaroslavl State University, Yaroslavl, 150003 Russia; 6 IM Sechenov First Moscow State Medical University, Moscow, 119146 Russia; 7 RUDN University, Moscow, 117198 Russia; 8 Federal Research Centre of Biological Systems and Agro-technologies of the Russian Academy of Sciences, Orenburg, 460000 Russia; 9 Nutrition Research Center, Taipei Medical University Hospital, 252 Wu-Hsing Street, Taipei, 11031 Taiwan

**Keywords:** Dietary patterns, Gender difference, Factor analysis, Obesity, Dyslipidemia, Abnormal fasting plasma glucose, Disease risk, Taiwan

## Abstract

**Background:**

The increasing prevalence of obesity has become a pandemic problem, and dietary patterns are one of the important factors causing obesity. Although the correlation between dietary patterns and obesity has been well explored, the gender difference on the association between dietary patterns and obesity remains unclear. The objective of this study examined whether gender difference existed in the relationship of dietary patterns with metabolic parameters and specific indices of adiposity among young and middle-aged adults with dyslipidemia and abnormal fasting plasma glucose (FPG) in Taiwan.

**Methods:**

A total of 14,087 subjects aged between 20 and 50 years with dyslipidemia and abnormal FPG were recruited in Taiwan between 2001 and 2010 for a cross-sectional study. Dyslipidemia was defined primarily according to the National Cholesterol Education Program Adult Treatment Panel III guidelines with minor modification. Abnormal FPG level was defined by the American Diabetes Association. Principal component analysis was conducted to identify dietary patterns. Multivariate logistic regression analysis was performed to evaluate the association of dietary patterns and metabolic parameters with different indices of adiposity including general obesity, central obesity, and high body fat, stratified by gender.

**Results:**

Two dietary patterns derived from principal component analysis were the prudent dietary pattern and the western dietary pattern. Both men and women in the highest quartile of the western dietary pattern had a significantly increased odds ratio of general obesity, central obesity, and high body fat. However, only male subjects in the higher quartiles of the prudent dietary pattern had a significantly decreased odds ratio of all indices of obesity. Both men and women with higher high-density lipoprotein cholesterol and total cholesterol levels had a significantly reduced odds ratio of general and central obesity, while those with higher triglycerides and FPG levels had a significantly increased odds ratio of general and central obesity. Higher low-density lipoprotein cholesterol level was significantly associated with an elevated odds ratio of high body fat, while higher total cholesterol level was significantly correlated with a reduced odds ratio of high body fat only in women.

**Conclusions:**

Gender difference exists in the association of dietary patterns and metabolic parameters with obesity and body fat in young and middle-aged adults with dyslipidemia and abnormal FPG in Taiwan.

## Background

Obesity has becoming a serious global health issue, affecting 23% of all Taiwanese [1], and is often associated with co-morbidities such as type 2 diabetes, cardiovascular and respiratory diseases, dyslipidemia, and some cancers. Abdominal obesity is the primary risk factor for cardiovascular disease and type 2 diabetes, and is particularly problematic in older adults [2, 3]. Obesity could reduce quality of life, cause substantial health complications, promote premature frailty, and accelerate age-associated declines in physical, cognitive, and mental functions [4]. It is estimated that by 2030 up to 57.8% of adults in the world would suffer from being overweight or obese [5]. The central causes of obesity include unhealthy dietary habits, lack of physical activity, and other environmental and behavioral factors. Past obesity research has investigated numerous correlates and causes of obesity, including demographic, genetic background, biological, medical, socioeconomic, and institutional factors [6–8].

Despite a large amount of efforts, the nutritional etiology of obesity remains controversial, especially with regards to possible roles of specific macronutrients (dietary fat and carbohydrate) or active compounds and gut microbiome in obesity. Some evidence suggests that dietary fiber found in whole grains and vegetables and saponins found in fruits may have a preventive role on obesity [9, 10]. Gut microbiota can influence energy utilization from food, glucose and lipid homeostasis, and immune response, and its profile in human specimen has shown to differ between obese and lean subjects [11]. It is known that dietary habits are the main contributor to the diversity of human gut microbiota by altering its composition [12]. Evidence has found that gut microbiota can influence body weight and percentage body fat [13]. Therefore, microbial changes in the human gut were proposed to be one of the possible causes of obesity [13].

Numerous studies have investigated the relationship between dietary patterns and certain diseases using factor analysis. Overall, dietary patterns reflect more accurate food consumption in daily life than studies of isolated nutrients, thus yielding results can offer greater insights into specific healthy dietary habits and public health recommendations [14]. These advantages have led to the analysis of dietary patterns being used widely to determine the association between diet and related chronic diseases in nutritional epidemiology in recent decades [15–18]. Furthermore, the type of weight gain correlated with specific dietary patterns and other factors should be explored further in order to understand the association between various measures of obesity and diseases such as type 2 diabetes, cardiovascular disease, certain cancers, and premature death [19–21].

Changes of dietary habits and decreased physical activity are considered the most important determining factors of overweight and obesity in Taiwan [22]. Furthermore, evidence had shown that gender differences affected the relationship between dietary patterns and obesity in Asian population [23]. The primary objective of this study was to examine whether gender difference existed in the relationship between dietary patterns and specific indices of adiposity such as general or central obesity and high body fat among young and middle-aged adults (aged between 20 and 50 y) with dyslipidemia and abnormal fasting plasma glucose (FPG) in Taiwan. The secondary objective of the study was to investigate gender difference in the association between metabolic parameters including high-density lipoprotein cholesterol (HDL-C), low-density lipoprotein cholesterol (LDL-C), total cholesterol (TC), triglycerides (TG), and FPG and indices of adiposity.

## Methods

### Subjects and study design

This cross-sectional study collected data from the Mei Jau (MJ) International Health Management Institution in Taiwan between 2001 and 2010. The MJ is a private health screening and management institution in Taiwan and consists of eight health screening centers in Asia, four of which are located in major cities (Taipei, Tayouan, Taichung, and Kaohsiung) in Taiwan. All subjects had their regular health check-up, were asked to complete a structured questionnaire which collected demographic data, lifestyle, dietary habits, anthropometric data, biochemical measures, and other health related data, and signed the informed consent form for research purpose only. Initially, the MJ database contained 765,064 adults who visited the MJ Health Management Institution for health screening between 2001 and 2010. A total of 96,088 subjects who were aged between 20 and 50 years and had both dyslipidemia and elevated fasting plasma glucose were retrieved. Subjects who had family history of diabetes and hyperlipidemia, with chronic diseases such as liver disorder, renal disorder, diabetes mellitus, or cancer, or on steroid, diabetic, thyroid, antivirus, or cardiovascular-related medication (*n* = 66,620) were excluded. After removing those with missing data (*n* = 15,381), the final samples of 14,087 subjects were analyzed in this study. This study was approved by the Taiwan Medical University Joint Institutional Review Board (N201810008).

### Definition of dyslipidemia and abnormal fasting plasma glucose

Based on the National Cholesterol Education Program Adult Treatment Panel III guideline [24] and the “cutoff” value for abnormal lipid levels in Taiwan [25], dyslipidemia was initially defined as meeting one of the following: (1) TG ≥ 2.3 mmol/L (200 mg/dL), (2) TC ≥ 6.2 mmol/L (240 mg/dL), or (3) LDL-C ≥ 4.1 mmol/L (160 mg/dL). Instead, our study used the modified definition of dyslipidemia as having the “borderline” high lipid levels: (1) TG ≥ 1.7 mmol/L (150 mg/dL), (2) TC ≥ 5.2 mmol/L (200 mg/dL), or (3) LDL-C ≥ 3.4 mmol/L (130 mg/dL). The purpose of using the modified definition of dyslipidemia was with the intention to spot those at higher risk for dyslipidemia earlier and provide timely interventions to delay the progression of cardiovascular disease. While FPG ≥ 5.6 mmol/L (100 mg/dL) was defined as abnormal blood glucose according to the diagnostic guidelines of American Diabetes Association [26].

### Assessment of dietary patterns

Dietary patterns were analyzed by using a standardized and validated semi-quantitative food frequency questionnaire (FFQ) developed by MJ Group [27, 28]. The FFQ contained 22 food groups featuring the typical consumption of Taiwanese food. The FFQ was designed to assess how frequently the participant consumed each food group with specific portion size in the past month (i.e. number of servings per day or per week from the lowest to the highest frequency). For instance, the consumption of milk was described as none or less than 1 glass/week, 1–3 glasses/week, 4–6 glasses/week, 1 glass/day, and 2 or more glasses/day (1 glass = 240 mL). For consumption of fruits and rice/flour products, 5 response options were available to choose from: < 1 serving/day, 1–2 servings/day, 2–3 servings/day, 3–4 servings/day, and ≥ 4 servings/day (1 serving = 1 medium-sized apple, 1 bowl of rice, or 2 bowls of noodles). As for consumption of light- or dark-colored vegetables, vegetables with oil or dressing, or root crops, the 5 response options were: < 0.5 bowls/day, 0.5–1 bowls/day, 1–1.5 bowls/day, 1.5–2 bowls/day, and 2 bowls/day (1 bowl = 11 cm in diameter). For intake of other food groups, the 5 response options were: < 1 serving/week, 1–3 servings/week, 4–6 servings/week, 1 serving/day, and ≥ 2 servings/day. Each question found in the FFQ had a clear definition on serving size for each food group consumed [28]. In this study, two dietary patterns were obtained from the FFQ using principal component analysis. We used eigenvalue ≥2 for the orthogonal rotation to derive dietary patterns and a factor loading ≥0.30 to categorize dietary patterns. The food group was considered in the dietary pattern with a higher factor loading value if the factor loadings of the food group were ≥ 0.30 in more than one dietary pattern. The factor score for each dietary pattern was calculated by using the sum of food consumption scores divided by factor loadings, and then each dietary pattern was further divided into quartiles [29].

### Anthropometric measurements

The assessments of anthropometric measurements were performed by the medical staff at the MJ health screening centers. Height (cm), weight (kg), and body fat (%) were measured by a bioelectrical impedance analysis instrument (InBody Co., Ltd., Seoul, South Korea). Body mass index (BMI) was calculated as weight (kg) divided by the square of height (m2), and further categorized as non-overweight, overweight, and obese status (BMI <  23 kg/m2 including individuals who were underweight and normal weight, BMI 23–24.9 kg/m2, and BMI ≥ 25 kg/m2, respectively) according to the modified classification of BMI for Asian population [30–34]. The waist circumference (WC) measurement was made at the approximate midpoint between the lower margin of the last palpable rib and the top of the iliac crest. In addition, hip circumference measurement was taken around the widest portion of the buttocks [19]. WC was defined as low (< 90 cm for men and < 80 cm for women) and high (≥ 90 cm for men and ≥ 80 cm for women) for Asians [30]. Waist-to-hip ratio (WHR) was calculated and defined as low (< 0.9 for men and <  0.8 for women) and high (≥ 0.9 for men and ≥ 0.8 for women) for Asians [30]. Waist-to-height ratio (WHtR) was defined as low (< 0.5) and high (≥ 0.5) for Asians [30]. Body fat (%) was defined as low (≤ 30% for men and ≤ 25% for women) and high (> 30% for men and > 25% for women) for Asian population [30].

### Biochemical profile

All blood samples were collected after overnight fasting for 12–14 h, and analyzed at the central laboratory of the MJ International Health Management Institution. Concentrations of serum HDL-C, TC, TG, and FPG were measured using the commercial reagents [28] and LDL-C concentrations were calculated using the Friedwald formula: LDL-C (mg/dL) = TC-(HDL-C + TG/5). The definitions of dyslipidemia and abnormal FPG used in this study were described previously.

### Covariates

Demographic and lifestyle characteristics including sex, age, marital status, education, current smoking, current drinking, and physical activity were collected by self-administered questionnaire from each subject. All subjects reported their marital status (single, married, or divorced), education (< university or ≥ university), current smoking (no or yes), current drinking (no: < 2 times/week or yes: ≥ 2 times/week), and physical activity (not active: < 150 min/week or active: ≥ 150 min/week) [28].

### Statistical analysis

Exploratory factor analysis (principal components) was used to identify dietary patterns on the basis of 22 food groups mentioned previously. Data adequacy for factor analysis had been confirmed by Kaiser-Meyer-Olkin measure of sample adequacy and Bartlett’s test of sphericity. Food groups were considered to contribute to the dietary pattern significantly if they had an absolute factor loading value ≥0.30. Subjects were categorized into quartiles according to the factor scores of each pattern. Quartile 1 (Q1) and quartile 4 (Q4) represented the lowest and the highest consumption of this dietary pattern, respectively. Results were expressed as the mean ± standard deviation (SD) for continuous variables and as percentages for categorical variables. For categorical data (sex, marital status, education, smoking, drinking, and physical activity), chi-square test was used to compare the differences in the characteristics of the subjects across different indices of adiposity. For continuous variables, one-way analysis of variance (ANOVA) and Bonferroni post-hoc test were used for comparison. Odds ratios (ORs) with 95% confidence intervals (CIs) were computed using multivariable logistic regression analysis to compare the association of dietary patterns and metabolic parameters with general obesity, central obesity, and high body fat, stratified by gender. Model 1 was the crude model, while model 2 was adjusted for age, marital status, education, smoking, drinking, and physical activity. For comparison of different quartiles of each dietary pattern, the first quartile (Q1) was considered as the reference group. Statistical analyses were performed using SPSS 23 (IBM Corp., Armonk, NY, USA), and *P* ≤ 0.05 was considered significant.

## Results

### Dietary patterns

The two dietary patterns derived from principal component analysis (Table 1) were named as western and prudent dietary patterns. The western dietary pattern was composed of higher intakes of deep-fried food, processed food (e.g. deli-meats such as ham, salami, sausages, and canned products), sauces (e.g. soy sauce, tomato sauce, hot sauce, salt, vinegar, pepper, and salad dressings), sugar-added beverages, instant noodles, meat, rice or flour cooked in oil (e.g. fried rice or noodles), organ meats (e.g. intestines, liver, heart, and kidneys), eggs, condiments like jam and honey, and refined dessert. On the other hand, a relatively healthier dietary pattern, the prudent dietary pattern, was described as containing higher intakes of dark- or light-colored vegetables (e.g. carrot, Chinese broccoli, broccoli, spinach, bok choy, tomato, cucumber, radish, cabbage, cauliflower, squash), fruits, vegetables with oil or dressing, root crops (e.g. potatoes, sweet potatoes, taro, Chinese yam, and corn), seafood, legumes and soy products (e.g. tofu, dried bean curd, and soybean milk), dairy products (e.g. liquid or powdered milk, cheese, and yogurt), milk, rice and flour products (e.g. rice, noodles, pasta, plain bread, and steamed buns), and whole grains (e.g. mixed grains, brown rice, whole wheat bread, multi-grain bread, and oats). In addition, rice/flour products, seafood, and legumes/soy products all had factor loadings greater than 0.30 in the two dietary patterns but were considered under prudent dietary pattern. Both refined dessert and eggs had factor loadings greater than 0.30 in the two dietary patterns but were considered under western dietary pattern (the factor loadings are shown in Table 1 in details). The western and prudent dietary patterns had a total variance of 36.3% (18.9 and 17.4%, respectively).

**Table 1:** Factor loadings and dietary patterns derived from principal component analysis

Food groups	Western dietary pattern	Prudent dietary pattern
Milk	0.044	0.421^a^
Dairy products	0.227	0.427^a^
Eggs	0.473^a^	0.328^a^
Meat	0.560^a^	0.275
Organ meats	0.510^a^	0.203
Legumes/soy products	0.362^a^	0.459^a^
Seafood	0.322^a^	0.463^a^
Light-colored vegetables	0.035	0.789^a^
Dark-colored vegetables	0.010	0.811^a^
Fruits	0.006	0.640^a^
Vegetables with oil/dressing	0.219	0.575^a^
Rice/flour products	0.346^a^	0.387^a^
Whole grains	0.161	0.381^a^
Root crops	0.262	0.544^a^
Refined dessert	0.441^a^	0.308^a^
Jam/honey	0.464^a^	0.208
Sugar-added beverages	0.601^a^	0.014
Rice/flour cooked in oil	0.513^a^	0.226
Deep-fried food	0.729^a^	0.093
Instant noodles	0.586^a^	0.033
Processed food	0.688^a^	0.117
Sauces	0.653^a^	0.043

^a^The values indicate a factor loading ≥0.30 used in the identification of dietary patterns

### Characteristics of the subjects

Characteristics of the subjects stratified by gender were shown in Table 2, while overall characteristics of the subjects across different indices of adiposity were described in Table 3. Men made up 75.9% of the subjects and were mostly from age group of 31–40 years (45.5%) and 41–50 years (42.0%), while women only contributed to 24.1% of the subjects and more than half (52.7%) of the women were aged between 41 and 50 years (Table 2). Most subjects in both genders were married (75.3% for men and 72.0% for women). More men (42.5%) reported having higher education (university or above) than women (25.5%). In terms of lifestyle, the majority of male subjects were non-smokers (63.7%) and non-drinkers (72.8%), while almost all of the female subjects were non-smokers (94.6%) and non-drinkers (94.1%). As for physical activity, the majority of men and women were inactive (81.0% for men and 86.6% for women).

**Table 2:** Characteristics of the subjects (*n* = 14,087)^a^

Variables	Total (*n* = 14,087)	Men (*n* = 10,691)	Women (*n* = 3396)	*P*^b^
Age, *n* (%)				< 0.001
20–30 y	1667 (11.8)	1332 (12.5)	335 (9.9)	
31–40 y	6139 (43.6)	4868 (45.5)	1271 (37.4)	
41–50 y	6281 (44.6)	4491 (42.0)	1790 (52.7)	
Marital status, *n* (%)				< 0.001
Single	3134 (22.3)	2417 (22.6)	717 (21.1)	
Married	10,497 (74.5)	8053 (75.3)	2444 (72.0)	
Divorced	456 (3.2)	221 (2.1)	235 (6.9)	
Education, *n* (%)				< 0.001
< University	8672 (61.6)	6142 (57.5)	2530 (74.5)	
≥ University	5415 (38.4)	4549 (42.5)	866 (25.5)	
Current smoking, *n* (%)				< 0.001
No	10,019 (71.1)	6805 (63.7)	3214 (94.6)	
Yes	4068 (28.9)	3886 (36.3)	182 (5.4)	
Current drinking, *n* (%)				< 0.001
No	10,984 (78.0)	7787 (72.8)	3197 (94.1)	
Yes	3103 (22.0)	2904 (27.2)	199 (5.9)	
Physical activity^c^, *n* (%)				< 0.001
Not active	11,605 (82.4)	8665 (81.0)	2940 (86.6)	
Active	2482 (17.6)	2026 (19.0)	456 (13.4)	
BMI, *n* (%)				< 0.001
< 23 kg/m^2^ (non-overweight)	4303 (30.6)	2600 (24.3)	1703 (50.1)	
23–24.9 kg/m^2^ (overweight)	3737 (26.5)	3059 (28.6)	678 (20.0)	
≥ 25 kg/m^2^ (obesity)	6047 (42.9)	5032 (47.1)	1015 (29.9)	< 0.001
WC, cm	82 ± 10	85 ± 8	74 ± 8	< 0.001
WHR	0.85 ± 0.07	0.87 ± 0.05	0.77 ± 0.05	< 0.001
WHtR	0.49 ± 0.05	0.50 ± 0.05	0.47 ± 0.05	< 0.001
Central obesity^d^, *n* (%)				
High WC	3478 (24.7)	2778 (26.0)	700 (20.6)	< 0.001
High WHR	3830 (27.2)	2950 (27.6)	880 (25.9)	0.055
High WHtR	5705 (40.5)	4903 (45.9)	802 (23.6)	< 0.001
High body fat^e^, *n* (%)	4640 (32.9)	1768 (16.5)	2872 (84.6)	< 0.001
HDL-C (mmol/L)	1.24 ± 0.49	1.16 ± 0.43	1.49 ± 0.55	< 0.001
LDL-C (mmol/L)	3.20 ± 1.14	3.19 ± 1.17	3.23 ± 1.05	0.063
TC (mmol/L)	5.56 ± 0.77	5.54 ± 0.78	5.61 ± 0.72	< 0.001
TG (mmol/L)	1.77 ± 0.85	1.90 ± 0.85	1.39 ± 0.74	< 0.001
FPG (mmol/L)	6.00 ± 0.98	6.01 ± 0.94	5.99 ± 1.10	0.312

*BMI* body mass index, *WHR* waist-to-hip ratio, *WC* waist circumference, *WHtR* waist-to-height ratio, *HDL-C* high-density-lipoprotein cholesterol, *LDL-C* low-density lipoprotein cholesterol, *TC* total cholesterol, *TG* triglycerides, *FPG* fasting plasma glucose

^a^Data are presented as the mean ± SD for continuous variables and *n* (%) for categorical variables

^b^*P*-values were derived from general linear regression for continuous variables and from chi-square test for categorical variables

^c^Not active < 150 min/week, active ≥150 min/week

^d^High WC ≥ 90 cm for men and ≥ 80 cm for women, high WHR ≥ 0.9 for men and ≥ 0.8 for women, high WHtR ≥0.5

^e^High body fat > 30% for men and > 25% for women

**Table 3:** Characteristics of the subjects across different indices of adiposity (*n* = 14,087)^a^

Variables	BMI (kg/m^2^)				WC^b^			WHR^c^			WHtR^d^			Body fat^e^		
	< 23 (*n* = 4303)	23–24.9 (*n* = 3737)	≥ 25 (*n* = 6047)	*P*^f^	Low (*n* = 10,609)	High (*n* = 3478)	*P*^f^	Low (*n* = 10,257)	High (*n* = 3830)	*P*^f^	Low (*n* = 8382)	High (*n* = 5705)	*P*^f^	Low (*n* = 9447)	High (*n* = 4640)	*P*^f^
Sex, *n* (%)				0.000			0.000			0.055			0.000			0.000
Men	2600 (60.4)	3059 (81.9)	5032 (83.2)		7913 (74.6)	2778 (79.9)		7741 (75.5)	2950 (77.0)		5788 (69.1)	4903 (85.9)		8923 (94.5)	1768 (38.1)	
Women	1703 (39.6)	678 (18.1)	1015 (16.8)		2696 (25.4)	700 (20.1)		2516 (24.5)	880 (23.0)		2594 (30.9)	802 (14.1)		524 (5.5)	2872 (61.9)	
Age, *n* (%)				0.000			0.000			0.000			0.000			0.005
20–30 y	583 (13.5)	379 (10.2)	705 (11.7)		1290 (12.1)	377 (10.8)		1403 (13.7)	264 (6.9)		1125 (13.4)	542 (9.5)		1117 (11.8)	550 (11.9)	
31–40 y	1917 (44.6)	1604 (42.9)	2618 (43.3)		4708 (44.4)	1431 (41.2)		4704 (45.9)	1435 (37.5)		3816 (45.5)	2323 (40.7)		4203 (44.5)	1936 (41.7)	
41–50 y	1803 (41.9)	1754 (46.9)	2724 (45.0)		4611 (43.5)	1670 (48.0)		4150 (40.4)	2131 (55.6)		3441 (41.1)	2840 (49.8)		4127 (43.7)	2154 (46.4)	
Marital status, *n* (%)				0.000			0.851			0.000			0.000			0.000
Single	1040 (24.2)	721 (19.3)	1373 (22.7)		2352 (22.2)	782 (22.5)		2417 (23.6)	717 (18.7)		1956 (23.3)	1178 (20.7)		2079 (22.0)	1055 (22.8)	
Married	3100 (72.0)	2892 (77.4)	4505 (74.5)		7917 (74.6)	2580 (74.2)		7519 (73.3)	2978 (77.8)		6137 (73.2)	4360 (76.4)		7150 (75.7)	3347 (72.1)	
Divorced	163 (3.8)	124 (3.3)	169 (2.8)		340 (3.2)	116 (3.3)		321 (3.1)	135 (3.5)		289 (3.5)	167 (2.9)		218 (2.3)	238 (5.1)	
Education, *n* (%)				0.003			0.000			0.000			0.000			0.000
< University	2624 (61.0)	2234 (59.8)	3814 (63.1)		6438 (60.7)	2234 (64.2)		6038 (58.9)	2634 (68.8)		5022 (59.9)	3650 (64.0)		5406 (57.2)	3266 (70.4)	
≥ University	1679 (39.0)	1503 (40.2)	2233 (36.9)		4171 (39.3)	1244 (35.8)		4219 (41.1)	1196 (31.2)		3360 (40.1)	2055 (36.0)		4041 (42.8)	1374 (29.6)	
Current smoking, *n* (%)				0.000			0.000			0.000			0.000			0.000
No	3290 (76.5)	2647 (70.8)	4082 (67.5)		7746 (73.0)	2273 (65.4)		7499 (73.1)	2520 (65.8)		6256 (74.6)	3763 (66.0)		6232 (66.0)	3787 (81.6)	
Yes	1013 (23.5)	1090 (29.2)	1965 (32.5)		2863 (27.0)	1205 (34.6)		2758 (26.9)	1310 (34.2)		2126 (25.4)	1942 (34.0)		3215 (34.0)	853 (18.4)	
Current drinking, *n* (%)				0.000			0.000			0.000			0.000			0.000
No	3532 (82.1)	2869 (76.8)	4583 (75.8)		8377 (79.0)	2607 (75.0)		8169 (79.6)	2815 (73.5)		6763 (80.7)	4221 (74.0)		7032 (74.4)	3952 (85.2)	
Yes	771 (17.9)	868 (23.2)	1464 (24.2)		2232 (21.0)	871 (25.0)		2088 (20.4)	1015 (26.5)		1619 (19.3)	1484 (26.0)		2415 (25.6)	688 (14.8)	
Physical activity ^g^, *n* (%)				0.010			0.007			0.403			0.227			0.000
Not active	3576 (83.1)	3018 (80.8)	5011 (82.9)		8687 (81.9)	2918 (83.9)		8433 (82.2)	3172 (82.8)		6932 (82.7)	4673 (81.9)		7552 (79.9)	4053 (87.3)	
Active	727 (16.9)	719 (19.2)	1036 (17.1)		1922 (18.1)	560 (16.1)		1824 (17.8)	658 (17.2)		1450 (17.3)	1032 (18.1)		1895 (20.1)	587 (12.7)	
Western dietary pattern, *n* (%)				0.000			0.000			0.061			0.000			0.000
Q1	1212 (28.1)	942 (25.2)	1367 (22.6)		2796 (26.3)	725 (20.8)		2616 (25.5)	905 (23.6)		2234 (26.7)	1287 (22.5)		2164 (32.9)	1357 (29.3)	
Q2	1148 (26.7)	970 (25.9)	1404 (23.2)		2712 (25.6)	810 (23.3)		2578 (25.1)	944 (24.7)		2155 (25.7)	1367 (24.0)		2376 (25.2)	1146 (24.7)	
Q3	1032 (24.0)	914 (24.5)	1576 (26.1)		2609 (24.6)	913 (26.3)		2537 (24.8)	985 (25.7)		2044 (24.4)	1478 (25.9)		2468 (26.1)	1054 (22.7)	
Q4	911 (21.2)	911 (24.4)	1700(28.1)		2492 (23.5)	1030 (29.6)		2526 (24.6)	996 (26.0)		1949 (23.3)	1573 (27.6)		2439 (25.8)	1083 (23.3)	
Prudent dietary pattern, *n* (%)				0.003			0.000			0.000			0.000			0.034
Q1	1032 (24.0)	901 (24.1)	1588 (26.3)		2584 (24.4)	937 (26.9)		2476 (21.1)	1045 (27.3)		2016 (24.0)	1505 (26.4)		2311 (24.5)	1210 (26.1)	
Q2	1038 (24.1)	946 (25.3)	1538 (25.4)		2602 (24.5)	920 (26.5)		2552 (24.9)	970 (25.3)		2051 (24.5)	1471 (25.8)		2346 (24.8)	1176 (25.3)	
Q3	1130 (26.3)	906 (24.3)	1486 (24.6)		2696 (25.4)	826 (23.7)		2622 (25.6)	900 (23.5)		2153 (25.7)	1369 (24.0)		2425 (25.7)	1097 (23.7)	
Q4	1103 (25.6)	984 (26.3)	1435 (23.7)		2727 (25.7)	795 (22.9)		2607 (25.4)	915 (23.9)		2162 (25.8)	1360 (23.8)		2356 (25.0)	1157 (24.9)	
HDL-C (mmol/L)	1.42 ± 0.57	1.21 ± 0.44	1.13 ± 0.41	0.000	1.28 ± 0.51	1.11 ± 0.39	0.000	1.27 ± 0.51	1.15 ± 0.40	0.000	1.32 ± 0.52	1.12 ± 0.40	0.000	1.20 ± 0.48	1.31 ± 0.50	0.000
LDL-C (mmol/L)	3.17 ± 1.14	3.22 ± 1.11	3.20 ± 1.15	0.282	3.21 ± 1.14	3.16 ± 1.13	0.055	3.20 ± 1.15	3.20 ± 1.11	0.722	3.20 ± 1.13	3.20 ± 1.15	0.922	3.18 ± 1.16	3.24 ± 1.10	0.002
TC (mmol/L)	5.59 ± 0.72	5.54 ± 0.76	5.55 ± 0.80	0.006	5.57 ± 0.75	5.52 ± 0.81	0.000	5.56 ± 0.75	5.54 ± 0.83	0.169	5.56 ± 0.74	5.55 ± 0.81	0.410	5.54 ± 0.77	5.60 ± 0.76	0.000
TG (mmol/L)	1.41 ± 0.74	1.81 ± 0.83	2.01 ± 0.85	0.000	1.67 ± 0.82	2.10 ± 0.85	0.000	1.66 ± 0.82	2.08 ± 0.86	0.000	1.58 ± 0.80	2.06 ± 0.85	0.000	1.80 ± 0.85	1.72 ± 0.85	0.000
FPG (mmol/L)	5.90 ± 0.98	5.95 ± 0.82	6.11 ± 1.06	0.000	5.94 ± 0.84	6.21 ± 1.29	0.000	5.91 ± 0.67	6.26 ± 1.50	0.000	5.90 ± 0.79	6.15 ± 1.19	0.000	5.97 ± 0.86	6.08 ± 1.18	0.000

*BMI* body mass index, *WC* waist circumference, *WHR* waist-to-hip ratio, *WHtR* waist-to-height ratio, *HDL-C* high-density-lipoprotein cholesterol, *LDL-C* low-density lipoprotein cholesterol, *TC* total cholesterol, *TG* triglycerides, *FPG* fasting plasma glucose

^a^Data are presented as the mean ± SD for continuous variables and *n* (%) for categorical variables

^b^Low WC < 90 cm for men and < 80 cm for women, high WC ≥ 90 cm for men and ≥ 80 cm for women

^c^Low WHR < 0.9 for men and < 0.8 for women, high WHR ≥ 0.9 for men and ≥ 0.8 for women

^d^Low WHtR < 0.5, high WHtR ≥0.5

^e^Low body fat ≤30% for men and ≤ 25% for women, high body fat > 30% for men and > 25% for women

^f^*P*-values were derived from general linear regression for continuous variables and from chi-square test for categorical variables

^g^Not active < 150 min/week, active ≥150 min/week

Speaking of obesity, the prevalence of overweight (BMI 23–24.9 kg/m^2^) was 28.6 and 20.0% among men and women, respectively. While the prevalence of general obesity (BMI ≥ 25 kg/m^2^) was 47.1 and 29.9% among men and women, respectively. In terms of central obesity, 26.0% men and 20.6% women had high WC, 27.6% men and 25.9% women had high WHR, and 45.9% men and 23.6% women had high WHtR. As for body fat, 16.5% men and 84.6% women had high body fat. In summary, the prevalence of general or central obesity was higher in men, while the prevalence of high body fat was higher in women in this selected population. For fasting blood lipid profile of the subjects, women had significantly higher HDL-C and TC levels but lower TG level than men.

Furthermore, subjects with obese BMI or central obesity were mostly men, aged 41–50 years, married, had low education level, low physical activity, low HDL-C, high TG, and high FPG levels (Table 3). While subjects with high body fat had similar characteristics as those who with obese BMI or central obesity, but were mostly women and had high HDL-C, high LDL-C, high TC, and low TG levels. Subjects with obese BMI or central obesity were mostly found in the highest quartile (Q4) of the western dietary pattern and the lowest quartile (Q1) of the prudent dietary pattern. While subjects with high body fat were mostly observed in the lowest quartile of the western dietary pattern.

### Dietary patterns, metabolic parameters, and indices of adiposity in men and women

The association of dietary patterns and metabolic parameters with general obesity, central obesity, and high body fat for both model 1 (crude data) and model 2 (adjusted for age, marital status, education, current smoking, current drinking, and physical activity) is shown in Tables 4 and 5. Both men and women in the highest quartile (Q4) of the western dietary pattern had a significantly increased odds ratio of general obesity (Table 4). However, only male subjects in the highest quartile (Q4) of the prudent dietary pattern had a significantly decreased odds ratio of general obesity. Furthermore, both men and women with higher HDL-C or TC level had a significantly reduced odds ratio of general obesity, while those with higher TG or FPG level had a significantly increased odds ratio of general obesity. However, LDL-C level had no significant correlation with general obesity.

**Table 4:** Odds ratios for general obesity and high body fat across quartiles of dietary pattern scores and metabolic parameters by gender^a^

Variables	OR (95% CI) for general obesity				OR (95% CI) for high body fat			
	Men (*n* = 10,691)		Women (*n* = 3396)		Men (*n* = 10,691)		Women (*n* = 3396)	
	Model 1^b^	Model 2^c^	Model 1^b^	Model 2^c^	Model 1^b^	Model 2^c^	Model 1^b^	Model 2^c^
Western dietary pattern								
Q2	0.96 (0.86, 1.07)	0.96 (0.86, 1.09)	1.08 (0.89, 1.30)	1.20 (0.99, 1.45)	0.89 (0.76, 1.05)	0.85 (0.72, 1.01)	1.14 (0.90, 1.44)	1.29 (1.01, 1.64)^*^
Q3	1.09 (0.98, 1.22)	1.10 (0.98, 1.23)	1.42 (1.16, 1.74)^***^	1.67 (1.36, 2.06)^***^	1.13 (0.97, 1.32)	1.00 (0.85, 1.17)	1.14 (0.88, 1.48)	1.43 (1.10, 1.88)^**^
Q4	1.28 (1.14, 1.42)^***^	1.27 (1.13, 1.43)^***^	1.30 (1.04, 1.62)^*^	1.66 (1.31, 2.09)^***^	1.56 (1.35, 1.80)^***^	1.26 (1.08, 1.47)^**^	1.04 (0.79, 1.37)	1.52 (1.14, 2.04)^**^
*P*-trend	0.000	0.000	0.001	0.000	0.000	0.000	0.579	0.002
Prudent dietary pattern								
Q2	0.91 (0.82, 1.01)	0.94 (0.84, 1.04)	1.08 (0.87, 1.33)	1.09 (0.88, 1.35)	0.89 (0.78, 1.02)	0.99 (0.86, 1.13)	1.06 (0.81, 1.39)	0.98 (0.75, 1.29)
Q3	0.88 (0.79, 0.98)^*^	0.92 (0.82, 1.02)	0.94 (0.76, 1.16)	0.96 (0.78, 1.20)	0.69 (0.60, 0.80)^***^	0.83 (0.72, 0.96)^*^	1.04 (0.80, 1.35)	0.93 (0.70, 1.22)
Q4	0.84 (0.75, 0.93)^**^	0.88 (0.79, 0.99)^*^	0.90 (0.73, 1.11)	0.92 (0.75, 1.15)	0.70 (0.61, 0.81)^***^	0.89 (0.77, 1.03)	1.07 (0.83, 1.39)	0.95 (0.72, 1.25)
*P*-trend	0.001	0.026	0.181	0.291	0.000	0.030	0.654	0.666
HDL-C (mmol/L)								
High	0.60 (0.55, 0.65)^***^	0.59 (0.54, 0.65)^***^	0.55 (0.44, 0.67)^***^	0.50 (0.40, 0.63)^***^	0.79 (0.71, 0.88)^***^	0.82 (0.73, 0.91)^***^	0.80 (0.59, 1.09)	0.65 (0.48, 0.89)^**^
*P*-trend	0.000	0.000	0.000	0.000	0.000	0.000	0.158	0.008
LDL-C (mmol/L)								
High	0.94 (0.88, 1.02)	0.96 (0.89, 1.03)	1.07 (0.92, 1.24)	1.01 (0.87, 1.18)	1.01 (0.91, 1.11)	1.07 (0.97, 1.19)	1.50 (1.24, 1.81)^***^	1.38 (1.13, 1.67)^**^
*P*-trend	0.138	0.257	0.380	0.874	0.917	0.180	0.000	0.001
TC (mmol/L)								
High	0.86 (0.79, 0.93)^***^	0.87 (0.80, 0.94)^***^	0.56 (0.47, 0.67)^***^	0.55 (0.47, 0.66)^***^	1.04 (0.93, 1.17)	1.10 (0.99, 1.23)	0.53 (0.40, 0.69)^***^	0.51 (0.39, 0.67)^***^
*P*-trend	0.000	0.001	0.000	0.000	0.449	0.089	0.000	0.000
TG (mmol/L)								
High	2.01 (1.86, 2.17)^***^	1.98 (1.83, 2.15)^***^	3.53 (3.01, 4.13)^***^	3.48 (2.97, 4.09)^***^	1.88 (1.69, 2.10)^***^	1.88 (1.68, 2.10)^***^	5.10 (3.73, 6.96)^***^	4.93 (3.60, 6.76)^***^
*P*-trend	0.000	0.000	0.000	0.000	0.000	0.000	0.000	0.000
FPG (mmol/L)								
High	2.39 (1.96, 2.91)^***^	2.34 (1.92, 2.86)^***^	3.39 (2.44, 4.73)^***^	2.95 (2.11, 4.13)^***^	2.29 (1.86, 2.81)^***^	2.61 (2.11, 3.23)^***^	2.65 (1.39, 5.07)^**^	2.18 (1.13, 4.20)^*^
*P*-trend	0.000	0.000	0.000	0.000	0.000	0.000	0.003	0.020

*HDL-C* high-density-lipoprotein cholesterol, *LDL-C* low-density lipoprotein cholesterol, *TC* total cholesterol, *TG* triglycerides, *FPG* fasting plasma glucose

^a^General obesity was defined as obese BMI (≥ 25 kg/m^2^), and high body fat was defined as > 30% for men and > 25% for women. The variables were defined as the following: HDL-C low < 1.04 mmol/L and high ≥1.04 mmol/L, LDL-C low < 3.4 mmol/L and high ≥3.4 mmol/L, TC low < 5.2 mmol/L and high ≥5.2 mmol/L, TG low < 1.7 mmol/L and high ≥1.7 mmol/L, and FPG low < 7.0 mmol/L and high ≥7.0 mmol/L. The lowest quartile (Q1) of the dietary pattern or low values of the variables were the reference group (not shown)

^b^Model 1 crude model

^c^Model 2 adjusted for age, marital status, education, current smoking, current drinking, and physical activity

^*^P < 0.05, ^**^P < 0.01, ^***^P < 0.001

**Table 5:** Odds ratios for central obesity across quartiles of dietary pattern scores and metabolic parameters by gender^a^

Variables	OR (95% CI) for central obesity (high WC)				OR (95% CI) for central obesity (high WHR)				OR (95% CI) for central obesity (high WHtR)			
	Men (*n* = 10,691)		Women (*n* = 3396)		Men (*n* = 10,691)		Women (*n* = 3396)		Men (*n* = 10,691)		Women (*n* = 3396)	
	Model 1^b^	Model 2^c^	Model 1^b^	Model 2^c^	Model 1^b^	Model 2^c^	Model 1^b^	Model 2^c^	Model 1^b^	Model 2^c^	Model 1^b^	Model 2^c^
Western dietary pattern												
Q2	1.09 (0.96, 1.25)	1.10 (0.96, 1.26)	1.23 (0.99, 1.53)	1.36 (1.09, 1.69)^***^	1.03 (0.91, 1.17)	1.09 (0.95, 1.24)	1.11 (0.91, 1.35)	1.26 (1.03, 1.54)^*^	1.01 (0.90, 1.13)	1.06 (0.94, 1.19)	1.08 (0.88, 1.32)	1.23 (1.00, 1.51)
Q3	1.24 (1.09, 1.41)	1.26 (1.10, 1.43)^***^	1.53 (1.22, 1.92)^***^	1.77 (1.40, 2.24)^***^	1.07 (0.94, 1.21)	1.19 (1.05, 1.36)^**^	1.27 (1.03, 1.56)	1.54 (1.24, 1.91)^***^	1.06 (0.95, 1.18)	1.17 (1.04, 1.31)^***^	1.29 (1.04, 1.61)^**^	1.56 (1.25, 2.95)^***^
Q4	1.48 (1.30, 1.68)^***^	1.50 (1.31, 1.71)^***^	1.63 (1.27, 2.08)^***^	2.04 (1.58, 2.64)^***^	1.12 (0.99, 1.26)	1.34 (1.18, 1.53)^***^	1.08 (0.86, 1.37)	1.48 (1.16, 1.90)^***^	1.16 (1.04, 1.29)^**^	1.35 (1.20, 1.52)^***^	1.25 (0.98, 1.58)	1.69 (1.32, 2.17)^***^
*P*-trend	0.000	0.000	0.000	0.000	0.053	0.000	0.167	0.000	0.004	0.000	0.015	0.000
Prudent dietary pattern												
Q2	0.94 (0.83, 1.06)	0.97 (0.86, 1.09)	1.14 (0.90, 1.45)	1.16 (0.91, 1.48)	0.89 (0.79, 1.00)^*^	0.89 (0.79, 1.00)	0.94 (0.75, 1.17)	0.94 (0.75, 1.18)	0.95 (0.85, 1.06)	0.94 (0.85, 1.05)	1.02 (0.81, 1.28)	1.03 (0.82, 1.30)
Q3	0.80 (0.71, 0.90)^***^	0.84 (0.74, 0.95)^**^	1.05 (0.83, 1.33)	1.08 (0.85, 1.38)	0.79 (0.70, 0.89)^***^	0.77 (0.68, 0.87)^***^	0.90 (0.72, 1.12)	0.90 (0.71, 1.13)	0.84 (0.76, 0.94)^***^	0.82 (0.73, 0.91)^***^	0.89 (0.71. 1.12)	0.91 (0.72, 1.16)
Q4	0.79 (0.70, 0.89)^***^	0.84 (0.74, 0.95)^***^	0.91 (0.72, 1.15)	0.93 (0.73, 1.19)	0.79 (0.70, 0.89)^***^	0.77 (0.70, 0.87)^***^	0.99 (0.80, 1.22)	0.98 (0.78, 1.23)	0.84 (0.76, 0.94)^**^	0.81 (0.73, 0.91)^**^	0.94 (0.75, 1.17)	0.95 (0.76, 1.20)
*P*-trend	0.000	0.001	0.305	0.441	0.000	0.000	0.834	0.801	0.000	0.000	0.336	0.476
HDL-C (mmol/L)												
High	0.59 (0.54, 0.65)^***^	0.58 (0.53, 0.64)^***^	0.51 (0.41, 0.65)^***^	0.48 (0.38, 0.60)^***^	0.66 (0.60, 0.72)^***^	0.61 (0.55, 0.67)^***^	0.64 (0.51, 0.79)^***^	0.54 (0.43, 0.67)^***^	0.59 (0.54, 0.65)^***^	0.56 (0.51, 0.61)^***^	0.59 (0.47, 0.74)^***^	0.52 (0.41, 0.66)^***^
*P*-trend	0.000	0.000	0.000	0.000	0.000	0.000	0.000	0.000	0.000	0.000	0.000	0.000
LDL-C (mmol/L)												
High	0.79 (0.73, 0.86)^***^	0.80 (0.74, 0.88)^***^	1.01 (0.86, 1.20)	0.96 (0.81, 1.14)	0.85 (0.78, 0.93)^***^	0.84 (0.77, 0.92)^***^	1.09 (0.94, 1.28)	1.01 (0.87, 1.19)	0.90 (0.84, 0.97)^**^	0.89 (0.83, 0.96)^**^	1.16 (0.99, 1.36)	1.09 (0.92, 1.28)
*P*-trend	0.000	0.000	0.881	0.647	0.000	0.000	0.253	0.874	0.007	0.004	0.069	0.313
TC (mmol/L)												
High	0.79 (0.72, 0.87)^***^	0.79 (0.72, 0.87)^***^	0.54 (0.45, 0.65)^***^	0.53 (0.44, 0.64)^***^	0.86 (0.79, 0.94)^**^	0.84 (0.77, 0.93)^***^	0.59 (0.50, 0.71)^***^	0.57 (0.47, 0.68)^***^	0.86 (0.79, 0.93)^***^	0.84 (0.77, 0.92)^***^	0.61 (0.51, 0.73)^***^	0.59 (0.49, 0.71)^***^
*P*-trend	0.000	0.000	0.000	0.000	0.000	0.000	0.000	0.000	0.000	0.000	0.000	0.000
TG (mmol/L)												
High	2.31 (2.10, 2.53)^***^	2.22 (2.02, 2.43)^***^	3.81 (3.20, 4.53)^***^	3.75 (3.15, 4.47)^***^	2.30 (2.10, 2.52)^***^	2.18 (1.98, 2.39)^***^	3.13 (2.66, 3.68)^***^	3.08 (2.61, 3.63)^***^	2.27 (2.10, 2.46)^***^	2.21 (2.04, 2.39)^***^	3.57 (3.02, 4.22)^***^	3.51 (2.97, 4.16)^***^
*P*-trend	0.000	0.000	0.000	0.000	0.000	0.000	0.000	0.000	0.000	0.000	0.000	0.000
FPG (mmol/L)												
High	3.06 (2.54, 3.69)^***^	2.91 (2.41, 3.52)^***^	5.20 (3.73, 7.25)^***^	4.66 (3.33, 6.52)^***^	3.36 (2.79, 4.06)^***^	2.77 (2.28, 3.36)^***^	8.05 (5.60, 11.56)^***^	7.11 (4.92, 10.27)^***^	2.91 (2.37, 3.56)^***^	2.53 (2.06, 3.11)^***^	5.57 (3.97, 7.80)^***^	4.82 (3.42, 6.79)^***^
*P*-trend	0.000	0.000	0.000	0.000	0.000	0.000	0.000	0.000	0.000	0.000	0.000	0.000

*WC* waist circumference, *WHR* waist-to-hip ratio, *WHtR* waist-to-height ratio, *HDL*-C high-density-lipoprotein cholesterol, *LDL-C* low-density lipoprotein cholesterol, *TC* total cholesterol, *TG* triglycerides, *FPG* fasting plasma glucose

^a^Central obesity was defined as high WC (≥ 90 cm for men and ≥ 80 cm for women), high WHR (≥ 0.9 for men and ≥ 0.8 for women), or high WHtR (≥ 0.5). The variables were defined as the following: HDL-C low < 1.04 mmol/L and high ≥1.04 mmol/L, LDL-C low < 3.4 mmol/L and high ≥3.4 mmol/L, TC low < 5.2 mmol/L and high ≥5.2 mmol/L, TG low < 1.7 mmol/L and high ≥1.7 mmol/L, and FPG low < 7.0 mmol/L and high ≥7.0 mmol/L. The lowest quartile (Q1) of the dietary pattern or low values of the variables were the reference group (not shown)

^b^Model 1 crude model

^c\^Model 2 adjusted for age, marital status, education, current smoking, current drinking, and physical activity

^*^P < 0.05, ^**^P < 0.01, ^***^P < 0.001

Both men and women in the higher quartiles (Q3 and Q4) of the western dietary pattern had a significantly increased odds ratio of central obesity in model 2 (Table 5). On the contrary, only male subjects in the higher quartiles (Q3 and Q4) of the prudent dietary pattern had a significantly decreased odds ratio of central obesity. Furthermore, all subjects with higher HDL-C or TC level had a significantly reduced odds ratio of central obesity. All subjects with higher TG or FPG level had a significantly increased odds ratio of central obesity. However, higher LDL-C level was significantly associated with a decreased odds ratio of central obesity only in men.

Both men and women in the highest quartile of the western dietary pattern had a significantly increased odds ratio of high body fat in model 2 (Table 4). However, only male subjects in the higher quartiles of the prudent dietary pattern had a significantly decreased odds ratio of high body fat. Men and women with higher HDL-C level had a significantly decreased odds ratio of high body fat in model 2. Higher LDL-C level was significantly associated with an increased odds ratio of high body fat, while higher TC level was significantly correlated with a decreased odds ratio of high body fat only in women. However, LDL-C or TC level had no significant association with high body fat in men.

## Discussion

The main finding of this study is that there is a gender difference in the association of dietary patterns and metabolic parameters with the risk of obesity and high body fat among young and middle-aged Taiwanese adults with dyslipidemia and abnormal FPG. Additionally, men and women who had greater consumption of a western dietary pattern increased the risk of general obesity, central obesity, and high body fat. However, only men who had greater consumption of a prudent dietary pattern decreased the risk of different indices of adiposity. The western dietary pattern in this study is comparable to the western pattern reported by Muga et al. [28] and Syauqy et al. [29], which included high intakes of processed food, meat, high-fat products, sauces, sweets, and sugary beverages. The EPIC-PANACEA (European Prospective Investigation into Cancer and Nutrition-Physical Activity, Nutrition, Alcohol, Cessation of Smoking, Eating Out of Home, and Obesity) study had similar findings that excess consumption of red meat, poultry, and processed meat might contribute to an increased risk of obesity [35]. The positive association between the western dietary pattern and the risk of obesity and high body fat can be explained by higher intake of energy-dense macronutrients such as fat and sugar and lower intake of beneficial nutrients such as dietary fiber, which is mostly found in fruits, vegetables, and whole grains [36, 37]. Our findings were consistent with a previous study which demonstrated a positive association between the western dietary pattern and different types of obesity in 486 Iranian women [38]. The prospective studies such as the Nurses’ Health study [39] and the study done by Slattery et al. [40] also concluded that a significant correlation between the western dietary pattern and elevated BMI. On the contrary, our study showed that a prudent diet featuring high consumption of vegetables, fruits, whole grains, legumes and soy products, milk and dairy products, and seafood was significantly associated with a decreased odds ratio of obesity and high body fat only in men. Our results were comparable to the third National Health and Nutrition Examination Survey data in US indicating that the prudent dietary pattern was significantly correlated with a lower likelihood of obesity only in males but not in females [41]. This finding in women was similar to two other previous studies, where Denova-Gutierrez et al. [42] found that there was no significant relationship between the prudent dietary pattern and the risk of general and central obesity, and Fung et al. [43] also reported no significant associations (*P* = 0.49) between the healthy dietary pattern and BMI. The association between dietary patterns and indices of adiposity is possibly attributed to the effect of dietary patterns on the composition of human gastrointestinal microbiota. The amount of energy extracted from food could be affected by the composition of the microbial communities in the gut. The two dominant beneficial bacterial phyla in human guts are *Bacteroidetes* and *Firmicutes*. Notably, different dietary patterns led to alteration in microbiome composition as shown by higher proportion of *Bacteroidetes* phylum in individuals consuming more plant-based diet compared to those on a western diet. Additionally, increased genera in *Firmicutes* phylum were observed in overweight and obese subjects, and thus *Firmicutes* phylum is considered to be important in the development of obesity [44]. The differences in the composition of microbiota confer the ability of microbiota in obese subjects to extract more calories from food than microbiota in lean subjects by breaking down indigestible polysaccharides [45]. Therefore, the manipulation of gut microbiota appears to be another approach in the treatment of obesity.

Our results indicated that some metabolic parameters such as HDL-C, LDL-C, TC, TG, and FPG were associated with obesity and high body fat. Additionally, higher HDL-C level was correlated with a decreased odds ratio of obesity and high body fat in both men and women. A previous study also found that HDL-C level was reduced in obese individuals, especially in the case of metabolic syndrome [46]. The Lipid Research Clinics Program Prevalence Study showed a significantly inversed association between BMI and plasma HDL-C level in children and adults of both sexes among 6865 white people [47]. Similarly, the third examination cycle of the Framingham Offspring Study revealed that BMI was negatively correlated with HDL-C concentration among 1566 men and 1627 women [48]. As BMI increased, there was a steady increase in TG level and a decline in HDL-C level [48]. Our findings are comparable to previous studies demonstrating that the negative correlation between HDL-C concentration and obesity seems to be stronger with central obesity, which is featured by visceral or intra-abdominal fat accumulation [49–51].

Evidences have shown that abdominal obesity was associated with high plasma TG and low plasma HDL-C [52, 53]. Our study also revealed that higher TG level was significantly correlated with an increased odds ratio of obesity and high body fat in both men and women. The possible mechanism of the positive association between TG and obesity is that insulin resistance, commonly found in individuals with abdominal obesity, promotes the atherogenic dyslipidemia characterized by elevated TG, low HDL-C, and high LDL-C levels [54]. Our results were consistent with the findings of a previous study which reported that subjects with higher serum TG and TC but low HDL-C levels were those who had overall and central obesity among women over 60 years. This may be due to high metabolic activity of the abdominal adipose tissue [55]. Similarly, individuals with elevated WC (abdominal obesity) had higher TG and TC levels in men and women [56]. Our results also suggest that women should have tighter control of their TG level because women with higher TG level appeared to have more than 3 times higher risk of general (OR = 3.48) or central obesity (OR = 3.08–3.75) and almost 5 times higher risk of high body fat (OR = 4.93) compared to those with lower TG level.

Inconsistent findings in our and other studies were noted for the association between plasma LDL-C and obesity (general or central) or high body fat. Our study found that higher LDL-C level had no correlation with general obesity in both men and women. In women, higher LDL-C level was not associated with central obesity, but was significantly correlated with an increased odds ratio of high body fat. However, in men, higher LDL-C level was inversely associated with central obesity, but had no correlation with high body fat. Our findings were not consistent with other studies. A Chinese cohort study reported that visceral fat accumulation was positively associated with serum LDL-C levels [57]. This association can be explained by the fact that high visceral fat accumulation is the major characteristic of central or abdominal obesity, which is an indicator of metabolic alterations with adverse outcomes such as hypertension, insulin resistance, hyperglycemia, reduced HDL-C level, and increased LDL-C and TG levels. One possible explanation for the inconsistent results may be due to different definitions: the definition of “high” LDL-C level in our study was ≥3.4 mmol/L unlike the definition from the National Cholesterol Education Program ATP III where high LDL-C level was defined as ≥4.1 mmol/L. The characteristics of male subjects with high LDL-C level in our study were mostly considered as having “borderline high” LDL-C level, being younger (mostly aged from 31 to 40 years), and also having normal WHR and WC. In contrast, enrolled subjects in other previous studies were often with a wider range of age (e.g. aged ≥45 years), and they followed the traditional guidelines to define “high” LDL-C level (≥ 4.1 mmol/L). The lipid profiles and their relation to disease risk could be influenced by age and the chosen definition for dyslipidemia.

Our study indicated that high FPG level was significantly associated with an increased odds ratio of obesity and high body fat for both men and women. Similar results were also found in another study which reporting a significantly positive correlation of fasting blood glucose with BMI and WC in outpatients in India [58]. Additionally, fasting blood glucose level was higher in overweight and obese children compared to that in normal children [59], and adolescents with high levels of overall and abdominal adiposities had the least favorable glucose level [60]. According to our study, higher FPG level appeared to have the strongest odds ratio with central obesity (OR = 4.66–7.11) compared to general obesity (OR = 2.95) and high body fat (OR = 2.18) in women. The increased intra-abdominal adipose tissue is the most clinically relevant type of body fat that was associated with metabolic complications such as insulin resistance and hyperglycemia in obese subjects [61]. Our results also suggest that women should aim for better control of their FPG level because women with higher FPG level appeared to have 5 to 7 times higher risk of central obesity compared to those with lower FPG level.

### Strengths and limitations

There are several strengths of this study. To the best of our knowledge, this is the first study to analyze the gender differences in the relationship between dietary patterns, metabolic parameters, and different indices of adiposity (e.g. general obesity, central obesity, and high body fat) in young and middle-aged Taiwanese adults with dyslipidemia and abnormal FPG. Moreover, the sample size was relatively large and comprised of a population of interest - the young and middle-aged adult population (aged 20 to 50 years). The findings remain unique because adults in this age group are the main supporters for the economics of the society, and their health status is worth special attention. Finally, unlike the previous studies, our study examined all indices of adiposity (BMI, WC, WHR, WHtR, and body fat) except body adiposity index. Although this study demonstrated significant gender differences in the association of dietary patterns with obesity (general and central) and body fat, several limitations still existed in this study. First, since this was a cross-sectional study, it did not address causality and therefore all the findings need to be confirmed in future prospective studies. Additionally, the potential residual confounding factor could not be removed completely as we did not consider subjects’ dietary behaviors (meal vs. snack patterns) in our dietary pattern analysis. Furthermore, the use of factor analysis often involves researchers’ bias, such as predetermination of the number of factors. Finally, the selection of subjects in this study was adults from a specific segment of the Taiwanese population: the working class who are mostly considered relatively healthy individuals. Thus, the conclusion could not apply to the entire Taiwanese population with dyslipidemia and abnormal FPG. Further prospective studies are needed to confirm the gender difference in the association of dietary patterns and metabolic parameters with different indices of adiposity.

## Conclusion

Our study showed that there is a gender difference in the relationship of dietary patterns and metabolic parameters with obesity (general and central) and body fat among young and middle-aged adults with dyslipidemia and abnormal FPG. The western dietary pattern is positively correlated with obesity (general and central) and high body fat in both men and women. However, the prudent dietary pattern is negatively correlated with obesity (general and central) and high body fat only in men. Higher LDL-C level is positively associated with high body fat, whereas higher TC level is negatively correlated with high body fat only in women. Overall, women are encouraged to have tighter control of their TG and FPG levels because they are highly related to an increased risk of central obesity.

## Data Availability

The data of the current study belong to the MJ International Health Management Institution and are restricted to the use of this study. The data are not publicly available. However, the data are accessible from the authors upon reasonable request and with the permission from MJ International Health Management Institution.

## Abbreviations

BMI: Body mass index
FFQ: Food frequency questionnaire
FPG: Fasting plasma glucose
HDL-C: High-density lipoprotein cholesterol
LDL-C: Low-density lipoprotein cholesterol
TC: Total cholesterol
TG: Triglycerides
WC: Waist circumference
WHR: Waist-to-hip ratio
WHtR: Waist-to-height ratio
